# Evaluating the effects of comprehensive simulation on central venous catheterization training: a comparative observational study

**DOI:** 10.1186/s12909-024-05661-2

**Published:** 2024-07-10

**Authors:** Haroula M. Tzamaras, Dailen Brown, Jessica Gonzalez-Vargas, Jason Moore, Scarlett R. Miller

**Affiliations:** 1 Department of Industrial Engineering, 307 Engineering Design and Innovation Building, Penn State, University Park, 16801 USA; 2 Department of Mechanical Engineering, Penn State, University Park, USA

**Keywords:** Comprehensive simulation, Medical simulation, Central venous catheterization

## Abstract

**Background:**

Simulation-based training (SBT) is vital to complex medical procedures such as ultrasound guided central venous catheterization (US-IJCVC), where the experience level of the physician impacts the likelihood of incurring complications. The Dynamic Haptic Robotic Trainer (DHRT) was developed to train residents in CVC as an improvement over manikin trainers, however, the DHRT and manikin trainer both only provide training on one specific portion of CVC, needle insertion. As such, CVC SBT would benefit from more comprehensive training. An extended version of the DHRT was created, the DHRT + , to provide hands-on training and automated feedback on additional steps of CVC. The DHRT + includes a full CVC medical kit, a false vein channel, and a personalized, reactive interface. When used together, the DHRT and DHRT + systems provide comprehensive training on needle insertion and catheter placement for CVC. This study evaluates the impact of the DHRT + on resident self-efficacy and CVC skill gains as compared to training on the DHRT alone.

**Methods:**

Forty-seven medical residents completed training on the DHRT and 59 residents received comprehensive training on the DHRT and the DHRT + . Each resident filled out a central line self-efficacy (CLSE) survey before and after undergoing training on the simulators. After simulation training, each resident did one full CVC on a manikin while being observed by an expert rater and graded on a US-IJCVC checklist.

**Results:**

For two items on the US-IJCVC checklist, “verbalizing consent” and “aspirating blood through the catheter”, the DHRT + group performed significantly better than the DHRT only group. Both training groups showed significant improvements in self-efficacy from before to after training. However, type of training received was a significant predictor for CLSE items “using the proper equipment in the proper order”, and “securing the catheter with suture and applying dressing” with the comprehensive training group that received additional training on the DHRT + showing higher post training self-efficacy.

**Conclusions:**

The integration of comprehensive training into SBT has the potential to improve US-IJCVC education for both learning gains and self-efficacy.

## Background

For over a decade, simulation-based training (SBT) has been integrated into medical education because it is an imitation of real-life events and procedures that can provide hands-on practice [1] without putting patients at risk [2]. One procedure that has seen an increase in the use of SBT is central venous catheterization (CVC). CVC involves the insertion of a catheter for quick and efficient medication delivery through the heart [3, 4], and over five million CVCs are conducted annually in the United States [3]. To conduct US-IJCVC, a series of steps must be followed in order. Table 1 illustrates the required steps of the procedure, as outlined by the New England Journal of Medicine [5]. The steps can be broken down into four main categories including procedural preparation, needle insertion, catheter placement, and post-catheter insertion and monitoring.

**Table 1:** Breakdown of the CVC steps and which are taught in the DHRT and the DHRT +

*Category*	*Main Steps of CVC*	*DHRT*	*DHRT* +
*Procedural Preparation*	Verbalize consent, universal precautions, and time out	**—**	**—**
	Preparing catheter kit: flushing catheter and checking equipment	**—**	**—**
	Maintaining sterile technique	**—**	**—**
	Selecting site for insertion	**✓**	**—**
	Injecting local anesthesia	**—**	**—**
*Ultrasound-Guided Needle Insertion*	Select correct ultrasound probe and use correct orientation	**✓**	**—**
	Obtaining clear image of target vessels using ultrasound	**✓**	**—**
	Correctly distinguishing between the vein and the artery	**✓**	**—**
	Inserting introducer needle at 35–45°	**✓**	**✓**
	Locating the needle’s position on the ultrasound	**✓**	**—**
	Advancing the introducer needle	**✓**	**✓**
	Achieving venous access	**✓**	**✓**
	Confirming vessel entry with needle aspiration	**✓**	**—**
*Catheter Insertion*	Removing syringe while occluding hub	**—**	**✓**
	Inserting guidewire into needle and advances without resistance	**—**	**✓**
	Maintaining control of the guidewire	**—**	**✓**
	Removing introducer needle	**—**	**✓**
	Using scalpel to make skin incision	**—**	**✓**
	Inserting and removes dilator	**—**	**✓**
	Passing catheter into vessel and removes wire	**—**	**✓**
	Inserting catheter to correct distance (14-17 cm)	**—**	**✓**
*Post-catheter insertion and monitoring*	Aspirating blood through the catheter	**—**	**✓**
	Securing catheter into place with suture and dressing	**—**	**✓**
	Placing order for an X-ray and monitoring catheter	**—**	**✓**

CVC is associated with high rates of complication [3, 6], which have been found to be directly correlated to the experience level of the person conducting the procedure [3, 4, 7]. A physician who has performed less than 50 catheterizations is two times more likely to incur complications than someone with more experience [4]. To reduce these complications, SBT is critical for providing more hands-on practice before performing CVC on patients [8]. The most common form of SBT used in CVC includes a manikin trainer [9] with a hand-pump arterial pulse and ultrasound guidance [3, 4]. Manikin simulators are useful for repetitive practice, but are manufactured to represent only a single patient anatomy, rely on the presence of trained observer to provide performance feedback to the learner, and degrade easily limiting what tools are allowed to be used with them [10, 11]. Moreover, manikin simulators mainly focus on needle insertion skills, and do not provide practice in all of the steps required for placing the catheter, including use of the guidewire, scalpel, dilator, and catheter, see Table 1. This training gap is crucial, since a lack of practice on these steps may increase the likelihood of mistakes among novice physicians’, such as arterial cannulation [4] or guidewire mismanagement [12].

**Fig. 1:**
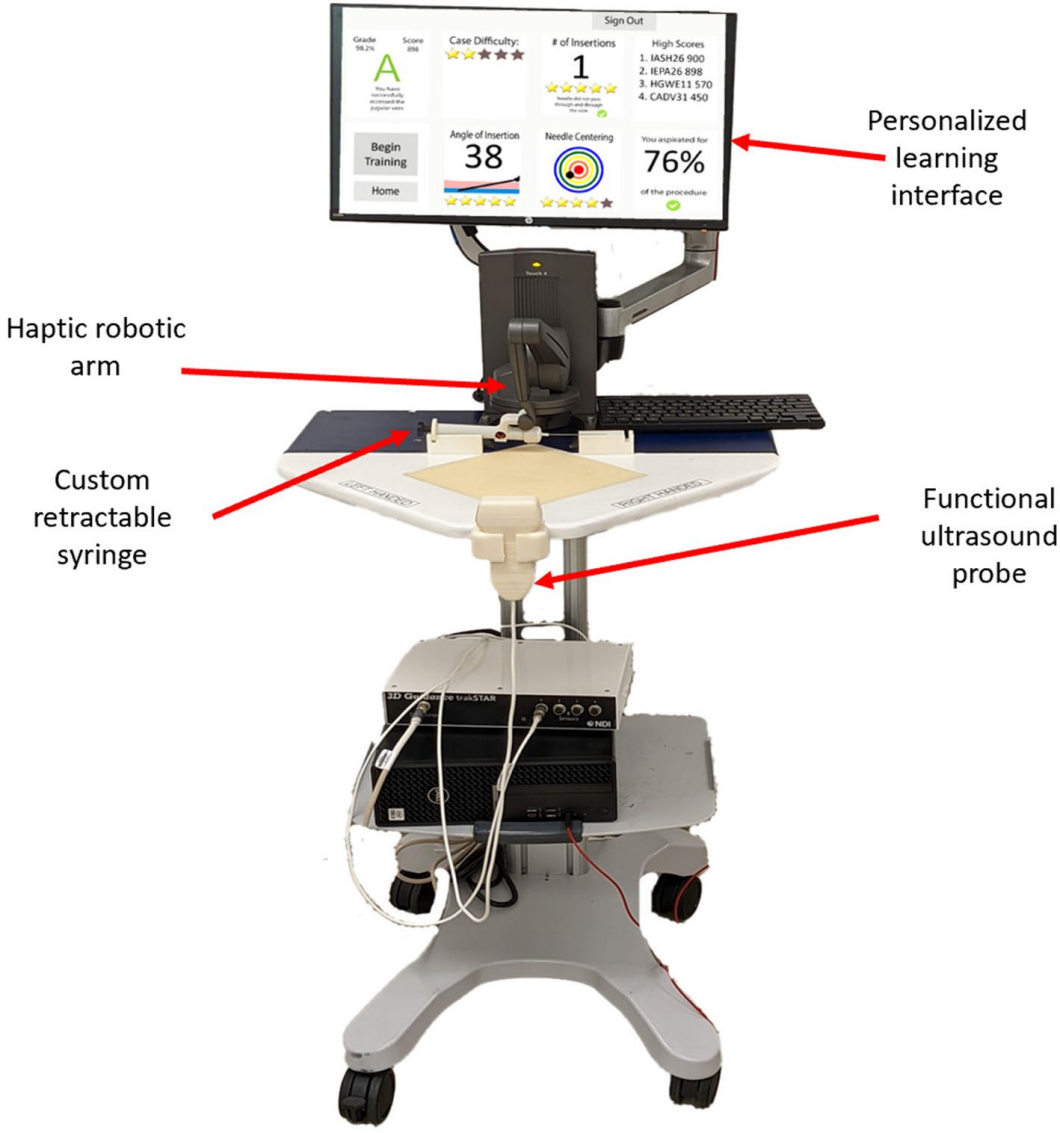
The DHRT system used for CVC training

To improve training for US-IJCVC training, researchers developed the Dynamic Haptic Robotic Trainer (DHRT), see Fig. 1 [13]. Specifically, the DHRT provides users with a step-by-step training of US-guided needle insertion [14]. The DHRT is made up of a haptic robotic arm, simulated ultrasound screen, and mock ultrasound probe [14], and includes 17 patient cases that differ based on the IJV size, depth, and location [15, 16]. In addition, the DHRT has a personalized learning interface [17] that provides automated performance feedback on various metrics including needle angle, number of insertion attempts, rate of aspiration, and needle centering [18] without the need for a trained observer. The DHRT was validated finding that is was as effective as manikin simulators based on a US-IJCVC checklist [19]. Previous studies on the DHRT have also indicated that self-efficacy, defined as confidence in oneself for specific tasks and outcomes [20], increases pre to post training [21]. Self-efficacy is important because evidence shows that performance and self-efficacy can be directly related, and can gauge the effectiveness of learning by the trainee [22].

**Fig. 2:**
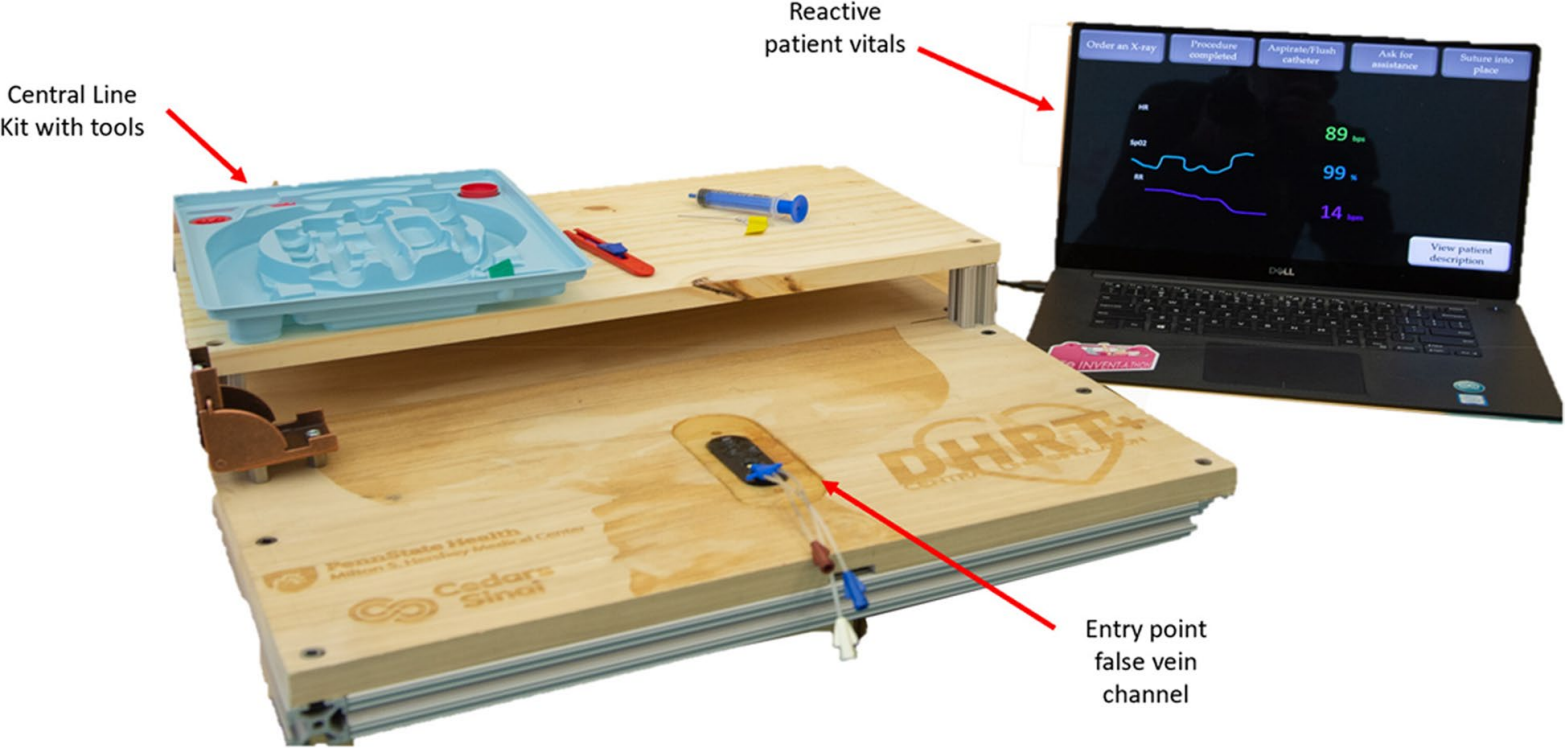
The DHRT + system (not pictured overhead camera for computer vision tracking)

While the DHRT has been proven to be an effective training method for needle insertion [19], it also focuses mainly on needle insertion.,. Prior work has demonstrated that comprehensive simulation training can increase residents’ experience level at a faster rate [23]. As such, we sought to develop the DHRT + . The DHRT + provides training on the steps of catheter insertion, see Table 1. Specifically, the DHRT + allows users to interact with a real CVC kit (e.g. guidewire, dilator, catheter, scalpel, and suture) and includes an interactive screen that provides patient vitals that react based on performance, see Fig. 2. The DHRT + also provides automated feedback by utilizing computer vision and a vein channel with sensors to track the order and accuracy of tool usage and relaying this information to the trainee on a graphical user interface post training. After inserting the catheter, the DHRT + requires trainees to use the interface to select the final steps of CVC in the appropriate order, including flushing and aspirating the catheter, suturing the catheter into place and dressing it, and ordering an X-ray.

When used in combination, the DHRT and DHRT + create a comprehensive training that allows trainees to practice with automated feedback, covering the critical needle insertion and catheter placement skills needed for US-IJCVC, see Table 1. Building on the foundations of previous work, the main objective of this study was to evaluate if the comprehensive training (the DHRT and DHRT +) impacted resident performance and self-efficacy compared to DHRT only training.

## Methods

Data for this study was collected at Hershey Medical Center (HMC) in the summers of 2021 and 2022 at new resident trainings. The remainder of this section highlights the methodology followed.

### Research questions

Specifically, the study was designed to answer the following research questions (RQ)s:

- RQ1: Is there a difference in performance on a US-IJCVC checklist between residents with comprehensive training on the DHRT + and residents trained only on the DHRT?
- The first research question was developed to determine if adding the DHRT + to training led to differences in performance between the two training groups according to expert-observed performance scores on a US-IJCVC checklist. The checklist can be divided into two categories: items that were only explicitly practiced through the DHRT + training (see Table 1), and items that were practiced by everyone. The primary hypothesis (H1) for this RQ was that residents in the comprehensive training group with the DHRT + who were exposed to hands-on practice inserting the catheter and going through more steps of the procedures would have more efficient movements and higher pass rates on the US-IJCVC checklist for items that were explicitly practiced on the DHRT + . This hypothesis is based on prior work in other fields of medical education that have indicated that focusing SBT on the whole procedure positively impacts learning gains and improves trainee performance [24, 25]. Secondarily, we hypothesized (H2) that there would be no differences in the remaining items as they were not trained differently between the two groups.
- RQ2: Is there a difference in self-efficacy between residents with comprehensive training on the DHRT + and residents trained only on the DHRT?
- The second research question was developed to determine if adding the DHRT + to training led to differences in self-efficacy between the two training groups, as measured by a central line self-efficacy (CLSE) survey. The CLSE can be divided into two categories: items that were only explicitly practiced through the DHRT + training (see Table 1), and items that everyone had the same exposure to. The primary hypothesis (H3) for this RQ was that all residents in both groups would increase self-efficacy pre to post training for all items but self-efficacy post training would be higher for the comprehensive training group for all items that were explicitly practiced through the DHRT + .. This is because prior research in other medical fields has indicated that more comprehensive exposure to simulation training can increase procedural confidence [26, 27], and more steps required for CVC [5] were covered with the comprehensive training. Secondly, we hypothesized (H4) that there would be no differences for the remaining items, as they were not trained differently between the two groups.

### Participants

A total of 106 medical residents at Hershey Medical Center participated in the study. In total, 42 participants identified as female, 63 as male, and one as other. Of all residents in this study, 14 were general surgery, 25 were anesthesia, 35 were internal medicine, 15 were emergency medicine, and the remainder were various other specialties, see Table 2.

**Table 2:** Summary of participant medical specialties

	**DHRT only**	**Comprehensive training group**	**TOTALS**
**Medical Specialty**			
*Acute Care*	0	1	1
*Anesthesiology*	12	13	25
*Emergency Medicine*	7	8	15
*General surgery*	0	14	14
*Internal medicine*	18	17	35
*Icu*	0	1	1
*Nephrology*	1	2	3
*Neurology*	3	1	4
*Ophthalmology*	2	0	2
*Pediatric critical care*	0	1	1
*Physical medicine and rehabilitation*	1	0	1
*Preliminary medicine*	1	0	1
*Pulmonary*	2	0	2
*Radiology*	0	1	1
**Total**	47	59	106

### Procedures

**Fig. 3:**
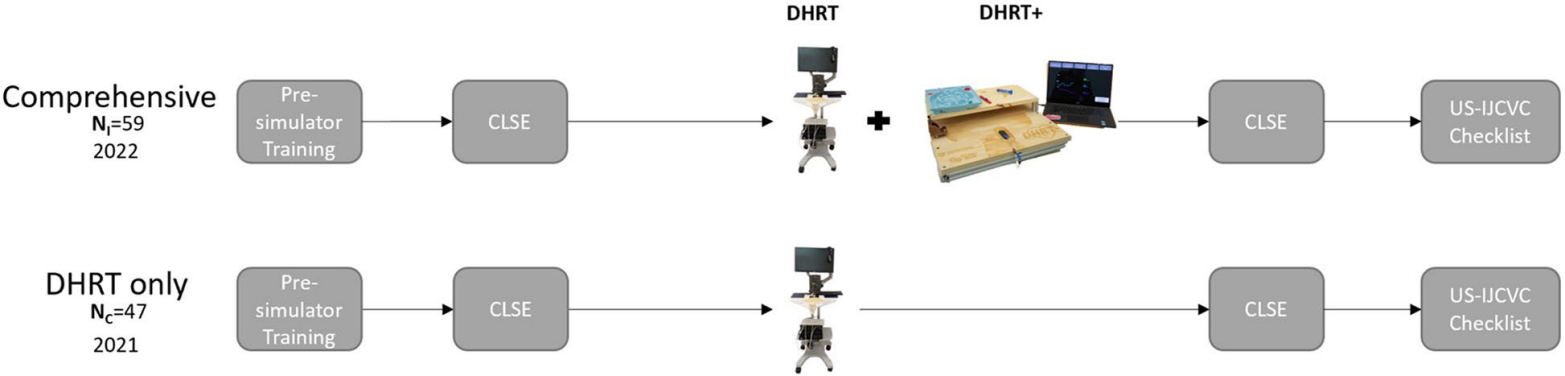
The training flow between the DHRT only and the comprehensive (DHRT and DHRT +) training groups

For all participants, informed consent was obtained according to an Institutional Review Board (IRB) approved protocol. Before coming to the in-person simulation training, all participants completed a pre-simulator online training including a demographic survey, a pre-test on CVC knowledge, eight interactive video modules covering CVC content, and a posttest on CVC knowledge, see [28] for more details on this training protocol. After completing the online training, residents were able to attend the in-person portion. Upon entering the simulation training, residents completed a 19-item 5-point Likert scale central line self-efficacy (CLSE) survey to assess their pre-training confidence on specific skills needed to successfully perform CVC. Next, all residents conducted trials on the DHRT system. In the DHRT only group in 2021, all residents conducted six trials on the DHRT and then went on to fill out the post training CLSE. In the comprehensive training group in 2022, the system was modified to include an interactive walkthrough of the procedure on the DHRT that showed residents how to use the simulator, and to modify the number of trials each resident completed on the DHRT based on performance. Residents who received a 70% or higher and avoided puncturing the carotid artery or backwall of the vein each trial were able to finish the training in as little as three trials, but could do up to six trials if more practice was needed. After the DHRT training, the comprehensive training group did one US-IJCVC on the DHRT + through to the final step of inserting the catheter and ordering and reading an X-ray. After using the DHRT + , the comprehensive training group filled out the post training CLSE form. Finally, in both training groups, each resident did one full US-IJCVC on a manikin trainer and were scored by an expert observer using a US-IJCVC checklist. The procedure and how it differed between training cohorts can be seen in Fig. 3.

### Metrics

The following metrics were used to assess differences between the comprehensive training (DHRT and DHRT +) group and the DHRT only group.

#### US-IJCVC checklist

The US-IJCVC checklist is a verification of proficiency checklist evaluated by an expert observer, someone who is trained on how to conduct the steps of the procedure. The US-IJCVC checklist is an assessment metric used to determine when a resident is proficient and can move from CVC simulation training to supervised CVC on patients [28]. The checklist includes one continuous variable, insertion attempts, and one ordinal variable, economy of time and motion. Insertion attempts is defined as how many tries it took with the needle before the resident was able to successfully insert it into the vein. Economy of time and motion is defined as the efficiency of the hand motion of the resident rated by an observer on a scale from 1–5 with 1 being the least efficient and 5 being the most efficient. The US-IJCVC checklist also includes 24 dichotomous items outlining all the mechanical and procedural steps and sub steps (refer to Table 1). For each dichotomous item, the observer would mark 1 for pass if the resident remembered to do the step *and* conducted it correctly or mark 0 for fail. If a resident passed every item on the checklist, they were considered competent in the procedure, otherwise they were recommended for further practice In addition to the breakdown of the 24 items, the economy of time and motion was considered as being explicitly practiced by the comprehensive training group, as the DHRT + allowed practice with the full range of tools needed for catheter insertion.

#### Central Line Self-Efficacy (CLSE) Survey

The 19-item 5-point Likert scale central line self-efficacy (CLSE) survey is used to assess confidence on skills needed to successfully perform CVC. These items include specific skills such as “locating vessels in an ultrasound image” or “securing the catheter with suture”, to more general skills such as “conducting the procedure without mistakes” or “conducting the entire procedure on a simulator”. The full CLSE survey can be found here.

#### Outcome measures

For RQ1, the outcome measures for the primary research question are economy of time and motion, and dichotomous checklist items including remove syringe, guidewire insertion, guidewire control, needle removal, verbalizes incision, verbalizes dilation, catheter insertion and wire removal, verbalizes catheter distance, and aspirates blood through catheter. For the secondary research question, the outcome measures are, insertion attempts and dichotomous checklist items including verbalizes consent, prepares kit, sterile technique, appropriate site, apply anesthesia, ultrasound orientation, ultrasound clear image, distinguish anatomy, needle insertion angle, locating needle on ultrasound, advancing needle, successful venipuncture, and confirm entry with aspiration.

For RQ2, the outcome measures for the primary research question were using the proper equipment in the proper order and securing the catheter with suture. The secondary outcome measures were verbalizing consent, preparing the catheter kit and equipment, obtaining clear image of the target vessel, locating the needle on the ultrasound, identifying the correct insertion site, using tactile feedback during placement, using ultrasound to identify the correct vessel, using tactile feedback to identify the vessel advancing the introducer needle, modifying the needle trajectory, identifying the needle in location, using tactile feedback to guide the needle, placing the needle in one attempt, placing the needle in multiple attempts, conducting the entire procedure without mistakes, conducting the entire procedure on a simulator, maintaining sterile technique.

### Data analysis

All analysis was conducted in SPSS (v. 29.0). To analyze differences on the US-IJCVC checklist, different statistical tests were run for each variable type. For the continuous variable, number of attempts, and the ordinal variable, economy of time and motion, Mann–Whitney U-tests were run to determine if there were differences between the DHRT and comprehensive training groups. To analyze differences in the 24 dichotomous pass/fail variables, a Pearson Chi-Square was used to test for significant differences in proportions. Fisher’s Exact Test was used in place of chi-square for any variable that did not have at least 5 residents fail in both the DHRT only and comprehensive training groups. All assumptions were met for both of these analyses.

To analyze differences in self-efficacy, a General Estimating Equation (GEE) was computed. Training group, CLSE type (pre or post-training), and their interaction were the independent variables and the CLSE questions were the dependent variables. All assumptions were met for GEE. For each variable with a significant interaction term, post hoc pairwise comparisons were conducted via an analysis of estimated marginal means.

## Results

The main objective of this research was to evaluate if the inclusion of comprehensive training (the DHRT and DHRT +) impacted resident performance and self-efficacy compared to the DHRT system alone. The following results are presented by research question.

### RQ1: Is there a difference in performance on a US-IJCVC checklist between residents with comprehensive training on the DHRT + and residents trained only on the DHRT?

For economy of time and motion, a Mann Whitney U test found no significant differences (*U* = 1466.5, *z* = 0.696, *p* = 0.486) between the DHRT (Md = 3) and the comprehensive training group (Md = 3) groups, partially refuting our primary hypothesis.. For the 24 dichotomous items on the US-IJCVC a Bonferroni correction was applied to account for repeated measures [29], resulting in a family-wise error rate adjusted alpha value of 0.002. One item practiced by the DHRT + group, “*aspirating blood through the catheter”* was significantly different (χ^2^ = 11.229, *p* < 0.001) between the proportion of residents who passed for this item in the comprehensive training group (81%) compared to the DHRT group (50%).For the continuous variable, insertion attempts, a Mann–Whitney U test found no significant differences (*U* = 1101.5*, z* = -0.401*, p* = 0.688) between the DHRT (Md = 1) and the comprehensive training group (Md = 1) groups. Of the remaining dichotomous variables, for “*verbalizing consent”*, there was a statistically significant difference (χ^2^ = 14.252, *p* < 0.001) between the proportion of residents who passed in the comprehensive training group (86.4%) compared to the DHRT group (53.2%). Full results from the Pearson Chi-Square and Fisher’s Exact test can be found in the Appendix.

### RQ2: Is there a difference in self-efficacy between residents with comprehensive training on the DHRT + and residents trained only on the DHRT?

To account for the repeated measures of the 19-item CLSE, a Bonferroni correction was applied [29], resulting in a family-wise error rate of 0.0026. GEE analysis revealed that the change from pre to post test was significant for all variables (*p* < 0.001). For the item “*securing the catheter with suture”* which was practiced on the DHRT + , the DHRT + group (Md = 4) rated higher (*Wald* χ^2^ = 16.343, *p* < 0.001), than the DHRT only group (Md = 3). For the item, “*using the proper equipment in the proper order”*, which also practiced on the DHRT + , the DHRT + group (Md = 4) also rated higher (*Wald* χ^2^ = 12.258, *p* < 0.001) than the DHRT only group (Md = 3). Additionally, there were significant interactions between the self-efficacy type (pre or post) and training group (DHRT or comprehensive) for one of the items on the CLSE survey, “*placing the needle in multiple attempts*” (*Wald* χ^2^ = 10.173, *p* = 0.001). Post hoc analysis via estimated marginal means (SE = 0.3403, p = 0.001, 95% CI [-1.752, -0.418]), revealed that while the pre-CLSE for this variable was significantly higher for the DHRT group than for the comprehensive training group (Mean difference = 0.51, *p* = 0.011), there were no significant differences after training. Full results from the GEE can be found in the Appendix.

## Discussion

The DHRT + system was developed because existing training methods used in US-IJCVC SBT focus on practicing the US-guided needle insertion portion of CVC (refer to Table 1) [10, 11], indicating a dire need to continuously create more comprehensive US-IJCVC education by covering more steps of the procedure. The main objective of this study was to evaluate if comprehensive training impacted resident self-efficacy and performance compared to training on the DHRT system alone. The main findings of this study indicated that.

- The comprehensive training group had better US-IJCVC checklist performance for verbalizing consent and aspirating blood through the catheter
- The comprehensive training group had higher self-efficacy for using the proper equipment in the proper order and securing the catheter with suture
- For all other items on the CLSE survey and the US-IJCVC checklist, comprehensive training was as effective as DHRT group since there were no significant differences between the groups

For the US-IJCVC checklist, we hypothesized that the residents in the comprehensive training group would perform better for items explicitly practiced on the DHRT + , including economy of time and motion (*H1)*. This hypothesis was based on prior literature, which indicated that exposure to a more comprehensive training with more steps of US-IJCVC covered would lead to more successful performance [25, 26]. Specifically, since the DHRT + included training in tool usage and equipment required for US-IJCVC for catheter placement, it was expected that the comprehensive training group would have more efficient hand motions. On the US-IJCVC, there no differences in economy of time and motion, refuting this part of our hypothesis. For the pass/fail items practiced on the DHRT + , only one, “*aspirating blood through the catheter”* was significantly different between groups, aligning with our hypothesis. As this is just one item of many, this significance does not conclusively prove the utility of including more comprehensive training, however, it may provide initial evidence that this is a viable and important teaching method for CVC. Additionally, for our secondary hypothesis (*H2)*, one item that was not explicitly practiced on the DHRT + , “*verbalizing consent”,* was also significantly higher for the comprehensive training group. This difference may have been due to residents thinking about the procedure as a whole since more steps were covered; however, further experimentation should be done to verify this effect of comprehensive training. For needle insertion attempts, and the other 22 pass/fail items on the US-IJCVC checklist, the DHRT only and the comprehensive training groups performed the same. Overall, these results partially support our hypotheses, and may indicate that a comprehensive CVC training with more steps of the procedure and automated performance feedback [11, 30] could be more effective for learning than trainers that focus only on needle insertion [24].

For the CLSE survey, we hypothesized (H3) that all residents would improve in self-efficacy for all items on the CLSE, but that self-efficacy post training would be higher for the comprehensive training group for items explicitly practiced on the DHRT + due to comprehensive training in more steps of the procedure covering more skills. We also hypothesized (H4) that self-efficacy for all other items would not be significantly different since these items were trained the same between groups. These hypotheses were based on prior literature indicating that SBT leads to confidence increases post training [21], and that more extensive procedural training can better increase procedural confidence [26, 27]. Our results indicated significant improvement from pre to post training for both groups, aligning with previous literature indicating the utility of SBT for US-IJCVC [21]. The comprehensive training group had significantly higher self-efficacy post training for both items on the survey that were explicitly practiced on the DHRT + , *“securing the catheter with suture*”, and *“using the proper equipment in the proper order”*. These results support our hypotheses that the inclusion of the DHRT + would positively impact resident self-efficacy. Ultimately, it was expected that there would be a larger increase in self-efficacy than what was observed; however, these initial findings may still indicate the utility of comprehensive training for improving self-efficacy.

Interestingly, although prior work has indicated that confidence and proficiency in surgical skills increase together [31], this was not the case for the comprehensive training. Specifically, self-efficacy items with higher ratings were not related to higher performance on the US-IJCVC checklist. For example, the residents in the comprehensive training group had a higher pass rate for *“verbalizing consent*”, but did not have higher self-efficacy for this item on the CLSE. These findings require further experimentation to determine if comprehensive training may overinflate resident confidence in their ability to perform parts of the procedure [32], as observed in prior work on medical residents and training [33]. Overall, the integration of a comprehensive training by adding the DHRT + training on the DHRT, shows initial potential for improving US-IJCVC education [34–36].

## Conclusion

The main objective of this paper was to evaluate if the integration of a comprehensive training impacted resident performance and self-efficacy. While the results of this study need to be further verified through future experimentation, we found initial evidence that the inclusion of comprehensive for CVC training could increase self-efficacy and checklist performance for several steps of the procedure. Future work should focus on validating these findings with a larger sample size, integrating the DHRT and the DHRT + into one comprehensive training tool instead of two separate devices used together, and determining why increases in self-efficacy and performance did not align.

There were some limitations of the study that must be addressed. First, due to the observational nature of this study, it is impossible to know conclusively from these results if the differences in the checklist and the survey were solely due to the intervention of the DHRT + . As such, follow-up experimentation with a larger sample size and additional outcome measures should be done to fully verify this finding. Secondly, for the US-IJCVC checklist and the self-efficacy survey, the data is filled on paper and is prone to human error and sections being skipped or missed. This led to small sample size changing slightly between variables if an observer missed a checkmark on the US-IJCVC; this can be observed in the results tables. Additionally, since they were multiple expert observers for the US-IJCVC checklist, there can be subjectivity in grading. Another limitation is the modification in required trials between training groups, which may have impacted self-efficacy in ways that were not evident from this study. Adding to this, data was collected at only one medical center in the United States which may limit the generalizability of these results. Finally, this study only exposed learners to one of two training conditions, the DHRT only and the DHRT + which adds additional training past the DHRT. Without including a third condition, it is impossible to know from this study alone if increases in self-efficacy and performance were due to the content of the DHRT + itself, or if the inclusion of any additional training would have made this difference. More experimentation would be needed to verify the findings of this study.

## Data Availability

The datasets used and/or analyzed during the current study are available from the corresponding author on reasonable request.

## Appendix

**Table 3:** Complete results of the chi-square analysis to compare pass rates on the dichotomous items from the US-IJCVC Checklist

Checklist Item	Treatment	Fail	Pass	Chi-square	*p*-value
*Verbalizes Consent*	Comprehensive	8 (13.6%)	51 (86.4%)	14.252	** < .001***
	DHRT	22(46.8%)	25 (53.2%)		
*Prepares Kit*	Comprehensive	15 (27.3%)	40(72.7%)	5.970	.015
	DHRT	23 (51.1%)	22(48.9%)		
*Sterile Technique*	Comprehensive	1 (1.7%)	57 (98.3%)	NA	.170
	DHRT	4 (8.5%)	43 (91.5%)		
*Appropriate Site*	Comprehensive	1 (1.7%)	57 (98.3%)	NA	1.00
	DHRT	1 (2.1%)	46 (97.6%)		
*Applied Anesthesia*	Comprehensive	7(12.1%)	51(87.9%)	2.514	.113
	DHRT	11(23.9%)	35(76.1%)		
*Ultrasound Orientation*	Comprehensive	3 (5.2%)	55 (94.8%)	NA	.462
	DHRT	5 (10.6%)	42 (89.4%)		
*Ultrasound Clear Image*	Comprehensive	0 (0%)	59 (100%)	NA	.194
	DHRT	2(4.3%)	45 (95.7%)		
*Distinguish Anatomy*	Comprehensive	2 (3.4%)	57 (96.6%)	NA	.502
	DHRT	0 (0%)	47 (100%)		
*Needle Insertion Angle*	Comprehensive	7(11.9%)	52(88.1%)	NA	.507
	DHRT	3 (6.4%)	44 (93.6%)		
*Locating Needle on Ultrasound*	Comprehensive	7 (12.1%)	51 (87.9%)	NA	.751
	DHRT	4 (8.5%)	43 (91.5%)		
*Advancing Needle*	Comprehensive	5 (8.6%)	53 (91.4%)	NA	.750
	DHRT	5 (10.6%)	42 (89.4%)		
*Successful Venipuncture*	Comprehensive	2 (3.6%)	53 (96.4%)	NA	.402
	DHRT	4 (9.1%)	40 (90.9%)		
*Confirm Entry with Aspiration*	Comprehensive	5 (8.5%)	54 (91.5%)	NA	1.000
	DHRT	3 (6.5%)	43 (93.5%)		
*Remove Syringe*	Comprehensive	22 (37.9%)	36 (62.1%)	1.284	.257
	DHRT	23 (48.9%)	24 (51.1%)		
*Guidewire Insertion*	Comprehensive	10 (17.5%)	47 (82.5%)	.044	.833
	DHRT	9 (19.1%)	38 (80.9%)		
*Guidewire control*	Comprehensive	8 (14%)	49 (86%)	.036	.850
	DHRT	6 (12.8%)	41 (87.2%)		
*Needle Removal*	Comprehensive	5 (8.8%)	52 (91.2%)	1.715	.190
	DHRT	8 (17.4%)	38 (82.6%)		
*Verbalizes Incision*	Comprehensive	1 (1.7%)	57 (98.3%)	7.752	.005
	DHRT	8 (17.0%)	39 (83.0%)		
*Verbalizes Dilation*	Comprehensive	1 (1.7%)	57 (98.3%)	NA	.170
	DHRT	4 (8.5%)	43 (91.5%)		
*Catheter Insertion and Wire Removal*	Comprehensive	4 (6.9%)	54 (93.1%)	NA	.723
	DHRT	4 (9.1%)	40 (90.9%)		
*Verbalizes Catheter Distance*	Comprehensive	14 (24.6%)	43 (75.4%)	6.517	.011
	DHRT	22 (48.9%)	23 (51.1%)		
*Aspirates Blood through Catheter*	Comprehensive	11 (19%)	47 (81.0%)	11.229	** < .001***
	DHRT	23 (50%)	423 (50%)		
*Verbalizes Suture*	Comprehensive	4 (6.9%)	54 (93.1%)	NA	.126
	DHRT	0 (0%)	47 (100%)		
*Verbalizes X-ray*	Comprehensive	2 (3.5%)	55 (96.5%)	NA	.135
	DHRT	6 (13.0%)	40 (87.0%)		

Fisher’s exact test was used for all chi-square columns of NA;** *** indicates significant *p* values for *p* < .002

**Table 4:** Complete results of the GEE for self-efficacy differences

Self-Efficacy Item	Predictor	Wald Chi-Square	df	*p*-value
*Obtaining clear image of the target vessel*	Treatment Group	.001	1	.981
	Pre or Post	65.789	1	** < .001***
	Interaction	3.547	1	.060
*Locating the needle on the ultrasound*	Treatment Group	.016	1	.899
	Pre or Post	51.103	1	** < .001***
	Interaction	.049	1	.826
*Identifying the correct insertion site*	Treatment Group	.133	1	.715
	Pre or Post	101.661	1	** < .001***
	Interaction	4.677	1	**.031***
*Using tactile feedback during placement*	Treatment Group	.010	1	.921
	Pre or Post	83.945	1	** < .001***
	Interaction	2.854	1	.091
*Using ultrasound to identify the correct vessel*	Treatment Group	.271	1	.603
	Pre or Post	81.086	1	** < .001***
	Interaction	1.093	1	.296
*Using tactile feedback to identify the vessel*	Treatment Group	.329	1	.566
	Pre or Post	74.557	1	** < .001***
	Interaction	.893	1	.345
*Advancing the introducer needle*	Treatment Group	.704	1	.401
	Pre or Post	57.448	1	** < .001***
	Interaction	1.451	1	.228
*Modifying the needle trajectory*	Treatment Group	.474	1	.491
	Pre or Post	69.721	1	** < .001***
	Interaction	.660	1	.417
*Identifying the needle in location*	Treatment Group	.066	1	.797
	Pre or Post	74.892	1	** < .001***
	Interaction	3.515	1	.061
*Using tactile feedback to guide the needle*	Treatment Group	.294	1	.588
	Pre or Post	60.026	1	** < .001***
	Interaction	5.123	1	**.024***
*Placing the needle in one attempt*	Treatment Group	.022	1	.883
	Pre or Post	98.672	1	** < .001***
	Interaction	.826	1	.363
*Placing the needle in multiple attempts*	Treatment Group	.489	1	.484
	Pre or Post	76.996	1	** < .001***
	Interaction	9.283	1	**.002***
*Conducting the entire procedure without mistakes*	Treatment Group	.067	1	.796
	Pre or Post	67.234	1	** < .001***
	Interaction	4.917	1	**.027***
*Conducting the entire procedure on a simulator*	Treatment Group	.184	1	.668
	Pre or Post	56.485	1	** < .001***
	Interaction	7.321	1	**.007***
*Verbalizing consent*	Treatment Group	4.848	1	**.028***
	Pre or Post	30.580	1	** < .001***
	Interaction	.363	1	.547
*Preparing the catheter kit and equipment*	Treatment Group	6.049	1	**.014***
	Pre or Post	27.353	1	** < .001***
	Interaction	.106	1	.744
*Maintaining sterile technique*	Treatment Group	7.686	1	**.006***
	Pre or Post	19.331	1	** < .001***
	Interaction	.106	1	.745
*Using the proper equipment in order*	Treatment Group	12.258	1	** < .001***
	Pre or Post	40.285	1	** < .001***
	Interaction	.255	1	.614
*Securing the catheter with suture*	Treatment Group	16.343	1	** < .001***
	Pre or Post	27.188	1	** < .001***
	Interaction	.001	1	.981

^*^indicates a significant value *p* < .0026

## Abbreviations

CVC: Central venous catheterization
US-IJCVC: Ultrasound guided central venous catheterization
DHRT: Dynamic Haptic Robotic Trainer
SBT: Simulation-Based Training
CLSE: Central Line Self-Efficacy Survey
GEE: General estimating Equation
